# Quantum Critical Scaling under Periodic Driving

**DOI:** 10.1038/s41598-017-06025-1

**Published:** 2017-07-18

**Authors:** Salvatore Lorenzo, Jamir Marino, Francesco Plastina, G. Massimo Palma, Tony J. G. Apollaro

**Affiliations:** 1 Dipartimento di Fisica e Chimica, Università degli Studi di Palermo, via Archirafi 36, I-90123 Palermo, Italy; 2 Quantum Technology Lab, Dipartimento di Fisica, Universita’ degli Studi di Milano, 20133 Milano, Italy; 3 Institute of Theoretical Physics, University of Cologne, D-50937 Cologne, Germany; 4 Dip. Fisica, Università della Calabria, 87036 Arcavacata di Rende (CS), Italy; 5 INFN - Gruppo collegato di Cosenza, Cosenza, Italy; 6 NEST, Istituto Nanoscienze-CNR, Pisa, Italy; 7 Centre for Theoretical Atomic, Molecular and Optical Physics, School of Mathematics and Physics, Queen’s University Belfast, Belfast, BT7 1NN United Kingdom; 8 INFN, Sezione di Milano, I-20133 Milano, Italy

## Abstract

Universality is key to the theory of phase transitions, stating that the equilibrium properties of observables near a phase transition can be classified according to few critical exponents. These exponents rule an universal scaling behaviour that witnesses the irrelevance of the model’s microscopic details at criticality. Here we discuss the persistence of such a scaling in a one-dimensional quantum Ising model under sinusoidal modulation in time of its transverse magnetic field. We show that scaling of various quantities (concurrence, entanglement entropy, magnetic and fidelity susceptibility) endures up to a stroboscopic time *τ*_*bd*_, proportional to the size of the system. This behaviour is explained by noticing that the low-energy modes, responsible for the scaling properties, are resilient to the absorption of energy. Our results suggest that relevant features of the universality do hold also when the system is brought out-of-equilibrium by a periodic driving.

## Introduction

A paradigm of phase transitions is the concept of universality, i.e., the insensitivity to microscopic details at the critical point of many particle systems at equilibrium. Universality allows to classify phase transitions according to critical exponents, which govern the scaling of several quantities close to the critical point. A quantum many body system at zero temperature can encounter a phase transition driven by quantum fluctuations when some of its control parameters are tuned to a critical value, which in the simplest case separates an ordered from a disordered phase^1^. As an hallmark of these quantum phase transitions (QPT), and in analogy with classical (temperature-driven) ones, physical observables exhibit scaling properties near such quantum critical point (QCP), with their leading behaviour depending only on few universal critical exponents^2, 3^. The insensitivity to microscopic physics and the emergence of universal critical exponents is in turn a consequence of the absence of any typical length scale in the system, as the correlation length diverges at criticality.

Temperature, however, plays a detrimental role in QPT, as it sets a thermal de Broglie length above which long-range correlations are suppressed^1^. This aspect renders the search for an analogue quantum critical behaviour in non-equilibrium (NEQ) many body systems a challenging task: an external agent pumping energy into the system^4–8^ induces an effective temperature delimiting the NEQ critical scaling region up to a characteristic ‘thermal’ length.

Among the rich varieties of NEQ drivings, perturbations periodic in time^9^ constitute a promising platform to engineer hopping in optical lattices^10–12^, artificial gauge fields^13, 14^, topological phases of matter^15–20^ as well as to induce dynamical localisation effects^21–24^. In general, a non-adiabatic perturbation heats a system^25^, and in absence of a bath dissipating the injected energy^24, 26^ or peculiar conditions–such as integrability^27, 28^ or disorder-induced localisation effects^29, 30^, a fully mixed, infinite temperature state will be eventually approached in the long-time dynamics. However, it has been shown that at intermediate time scales novel interesting effects can still be observed in an isolated periodically driven, ergodic system, such as the onset of NEQ long-lived metastable states^31–33^.

In this study, we consider the *resilience of critical scaling under periodic driving*. Specifically, we show the *robustness* of critical scaling exponents when a time-periodic modulation is super-imposed on the transverse magnetic field of a Quantum Ising model^28, 34, 35^ prepared in its critical ground state. Indeed, a number of quantities, evaluated on the time dependent out-of-equilibrium state of the periodically driven system, follows the same scaling^3^ behaviour proper of the equilibrium QCP, even though the state itself is very far from the critical ground state. This behaviour persists up to a stroboscopic time scale, *τ*_*bd*_, where scaling breaks down, thus setting a condition for the observation of quantum critical scaling in periodically driven many body systems.

## Results

### Periodically driven Ising model

We investigate the 1D quantum *XY*-model, driven by a periodic transverse magnetic field:

1 $$\hat{H}(t)=-\sum _{i=1}^{N}\,(\frac{1+\gamma }{2}{\hat{\sigma }}_{i}^{x}{\hat{\sigma }}_{i+1}^{x}+\frac{1-\gamma }{2}{\hat{\sigma }}_{i}^{y}{\hat{\sigma }}_{i+1}^{y}-h(t){\hat{\sigma }}_{i}^{z}),$$

where ${\hat{\sigma }}^{\alpha }$ ($\alpha =x,y,z$) are the Pauli matrices, $h(t)=h+{\rm{\Delta }}h\,\sin \,(\omega t)$ is the harmonically modulated transverse field, and *γ* the anisotropy parameter. For $\gamma \in (0,1]$, this model belongs to the Ising universality class and it exhibits a second order QPT with a critical point located at *h* = *h*_*c*_ = 1, separating a ferromagnetic phase from a paramagnetic one. The *XY*-model with a static field is diagonalised by standard Jordan-Wigner (JW) and Bogolyubov transformations^1, 36^, enabling Eq. (1) (with Δ*h* = 0) to be re-written as a free fermion Hamiltonian

2 $$\hat{H}=\sum _{k}\,{\varepsilon }_{k}(2{\hat{\gamma }}_{k}^{\dagger }{\hat{\gamma }}_{k}-1).$$

Here ${\varepsilon }_{k}=\sqrt{{(h-\cos k)}^{2}+{(\gamma \sin k)}^{2}}$ is the energy of the Bogolyubov quasiparticle with momentum *k*, and annihilation operator ${\hat{\gamma }}_{k}={u}_{k}{\hat{c}}_{k}+{v}_{k}{\hat{c}}_{-k}^{\dagger }$, the *c*_*k*_’s being JW spinless fermion operators labelled by the momentum *k*. The ground state of Eq. 2 can be written in the BCS form

3 $$|GS\rangle \equiv |{\rm{\Psi }}(t=\mathrm{0)}\rangle =\prod _{k > 0}\,{|\psi \mathrm{(0)}\rangle }_{k}=\prod _{k > 0}\,({u}_{k}+{v}_{k}{\hat{c}}_{k}^{\dagger }{\hat{c}}_{-k}^{\dagger })\,|0\rangle ,$$

with |0〉 representing the vacuum of the JW fermions (${\hat{c}}_{k}|0\rangle =\mathrm{0,}\forall k$). The ground state (3) at the critical point (*h* = *h*_*c*_) is the initial state for the periodic drive considered in this study.

Floquet analysis is a valuable tool to deal with time-periodic Hamiltonians, $\hat{H}(t)=\hat{H}(t+nT)$, as it allows to reduce the *stroboscopic* time evolution, i.e. at integer steps *n* of the period *T*, to a dynamics generated by a *time*-*independent* effective Hamiltonian^9^. Indeed, by exploiting the periodicity of $\hat{H}(t)$, the (stroboscopic) unitary time evolution operator $\hat{U}(t)$, at times *t* = *nT*, can be written as a discrete-time quantum map $\hat{U}(nT)={[\hat{U}(T)]}^{n}$, where the effective (Floquet) Hamiltonian, ${\hat{H}}_{F}$, is hence defined^27^ by $\hat{U}(T)={e}^{-iT{\hat{H}}_{F}}$. In the periodically driven XY model, the Floquet operator can be expressed as a product of operators acting in each two-dimensional *k*-subspace, spanned by the vacuum and by the state with a pairs of JW fermions $\{|{0}_{k}{0}_{-k}\rangle ,|{1}_{k}{1}_{-k}\rangle \}$, namely $\hat{U}(T)={\prod }_{k > 0}\,{\hat{U}}_{k}(T)$. Accordingly, after *n* periods of the sinusoidal drive, the initial critical state (3) evolves into $|{\rm{\Psi }}(nT)\rangle =\hat{U}(nT)|{\rm{\Psi }}\mathrm{(0)}\rangle ={\prod }_{k > 0}\,{\hat{U}}_{k}(nT){|\psi \mathrm{(0)}\rangle }_{k}$ (for further details see Suppl. Mat. of ref. 28).

$|{\rm{\Psi }}(nT)\rangle$ will be the stroboscopic state where we test the persistence of finite size scaling (FSS) behaviour, the characteristic trait of criticality, in several quantities and for a number of different driving conditions.

### Critical scaling under periodic drive

In the following, we provide evidence that the scaling behaviour of a number of physical quantities, which should strictly hold at the equilibrium QCP only, persists in fact also under periodic driving. In particular, we consider both local–in the real lattice space, quantities (e.g., nearest-neighbor concurrence and local transverse magnetic susceptibility) and non-local ones (e.g., entanglement entropy and fidelity susceptibility).

Remarkably, scaling behaviour at equilibrium has been found also for quantities that are not observables in the strict quantum mechanical sense, such as entanglement. Indeed, the concurrence^37^, a measure of bi-partite entanglement for two qubits (see Methods), has been shown to exhibit FSS at equilibrium QCP, as first demonstrated for the quantum Ising model^38^ and later illustrated in other systems^39–43^. The scaling of concurrence was explored in details, as it bridges QPT with quantum information theory^44, 45^ and quantum thermodynamics, because of its close connection with the notions of irreversible work^46^ and ergotropy^47^.

In the quantum Ising (QI) model at the equilibrium critical point^38^, the derivative of the concurrence between neighboring spins with respect to the transverse field, *h*, displays a logarithmic singularity at *h* = *h*_*c*_. An FSS analysis at equilibrium for the concurrence shows data collapse for different system sizes, consistent with the universal critical exponent $\nu =1$, which also governs the divergence of the correlation length of the order parameter in the QI model.

**Figure 1:**
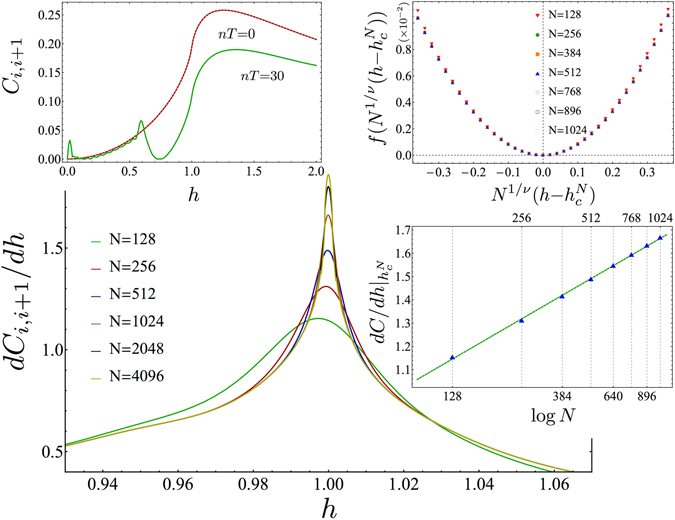
Finite-size scaling of the concurrence. Main (central) plot: $\frac{d{C}_{i,i+1}(N)}{dh}$ vs. initial magnetic field *h* around the QPT point for different system’s sizes *N*. A logarithmic divergent behaviour is found close to *h* = 1, as inferred from the right lower inset, where the maximum of the peaks of $\frac{d{C}_{i,i+1}(N)}{dh}$ vs *N* are reported in a semilogarithmic plot. Upper Left inset: Nearest-neighbor Concurrence *C*_*i*,*i*+1_(*N*) vs. initial magnetic field *h* at equilibrium, for an infinite chain (red-dotted line) and after *n* = 30 cycles for *N* = 128, 256, 512, 1024, 2048, 4096, (green continuous lines: different lines are not distinguishable on this scale). Upper right inset: Data collapse for FSS at the logarithmic divergence, for the same *N* as listed above, after *n* = 30 cycles, according to the Ansatz $1-\exp ({\frac{dC(N)}{dh}-\frac{dC(N)}{dh}|}_{h={h}_{c}^{N}})=f({N}^{\frac{1}{\nu }}(h-{h}_{c}^{N}))$, with $\nu =1$. The driving protocol is *h*(*t*) = 1 + 0.1 sin 2*πt*, and we took *γ* = 1 (cfr. Eq. 1). The chosen value of *n* = 30 is within the breakdown time of the shortest chain here considered (see following section). For *n* < 30, although the numerical values of $\frac{d{C}_{i,i+1}(N)}{dh}$ (and *C*_*i*,*i*+1_(*N*)) change, the FSS data collapse and the logarithmic divergence, upper right and left inset, respectively, is attainable with the same critical exponent.

Interestingly, we find that the same scaling property still holds for a periodically driven, out of equilibrium system. The nearest-neighbor concurrence *C*_*i*,*i*+1_(*N*) for different system sizes *N* is reported in the upper left inset of Fig. 1 both at equilibrium, *nT* = 0, and after time *nT* = 30 of the driving $h(t)=h+0.1\,\sin \,(2\pi t)$. Notice that, notwithstanding the system is driven out-of-equilibrium, the qualitative features of *C*_*i*,*i*+1_(*N*) around the critical point *h*_*c*_ are preserved. Indeed, in the main plot of Fig. 1, we report the derivative of the stroboscopic nearest-neighbor concurrence, $\frac{d{C}_{i,i+1}(N)}{dh}$, in the neighborhood of *h*_*c*_ for different system sizes *N* and after $n=t/T=30$ cycles of the driving $h(t)={h}_{c}^{N}+0.1\,\sin \,(2\pi t)$, where ${h}_{c}^{N}$ is the pseudocritical point, where ${h}_{c}^{N}$ is the pseudocritical point, which moves towards *h*_*c*_ as ${h}_{c}-{N}^{-2}(\mathrm{log}\,N+const)$. It is interesting to note that the logarithmic correction to the shift exponent *λ* = 2 is shared also by the equilibrium FSS behaviour of the half-chain entanglement entropy^48^. With increasing system’s size *N*, a logarithmic divergence in the concurrence builds up at $h={h}_{c}^{N}$, namely ${\frac{d{C}_{i,i+1}(N)}{dh}|}_{h={h}_{c}^{N}}\propto \,\mathrm{log}\,N$ (see the lower right inset of Fig. 1). Performing the FSS analysis for logarithmic divergences^3^, we obtain data collapse once the scaling exponent is set to its equilibrium value $\nu =1$, as reported in the upper right inset of Fig. 1. FFS is attained with the same value of the critical exponent $\nu$ even after changing driving amplitude, frequency, anisotropy parameter as well as number of cycles, provided the conditions outlined in the following subsection are fulfilled. This highlights that the role played by universality stretches well beyond the ground state properties, significantly affecting the system even under periodic driving.

In the following we will substantiate further our claim about the persistence of FSS in periodically driven critical systems by considering additional quantities. For this purpose, we have also considered the scaling relation for the transverse magnetic susceptibility of the *XY*-model, ${\chi }_{z}^{(N)}(h)=\frac{1}{N}\frac{d\langle {\hat{M}}^{z}\rangle }{dh}=\frac{d\langle {\hat{\sigma }}^{z}\rangle }{dh}$. At equilibrium, it exhibits a scaling behaviour with critical exponent *α* = 0^49^, implying a logarithmic divergence akin to the one encountered for the concurrence. Such logarithmic divergence is preserved also under driving, and, in analogy with the scaling Ansatz for the concurrence, data collapse is obtained for different system’s sizes, implying that even after the system is brought out-of-equilibrium by periodic driving, the scaling exponents keep their equilibrium values *α* = 0 and $\nu =1$. So far, we have considered single and two-site quantities. Still, physical quantities having support on a larger part of the system, e.g., the entanglement entropy, or even genuinely global, such as the Fidelity susceptibility, exhibit FSS at equilibrium in the critical QI model^48, 50^. The former following a logarithmic and the latter an algebraic divergence, respectively. As for the Entanglement Entropy of the half chain *S*_*N*/2_, defined by the von Neumann entropy ${S}_{N/2}=-{\rm{tr}}\,\{{\hat{\rho }}_{N/2}\,{\mathrm{log}}_{2}\,{\hat{\rho }}_{N/2}\}$, we find that the FSS relation ${S}_{N}(h)-{S}_{N/2}({h}_{c}^{N})=f({N}^{\frac{1}{\nu }}(h-{h}_{c}^{N}))$, based on the logarithmic law of the entanglement entropy at criticality, derived in ref. 48, holds as well under periodic driving up to times ${t}_{N\mathrm{/2}}=\frac{N}{2{v}_{max}}$, where *v*_*max*_ is the maximum group velocity of the Floquet quasiparticles^51–53^ (see Methods). Indeed *t*_*N*/2_ gives a time after which the quasi-particles have left the half chain and a volume law for the Entanglement Entropy is attained^51, 54, 55^.

**Figure 2:**
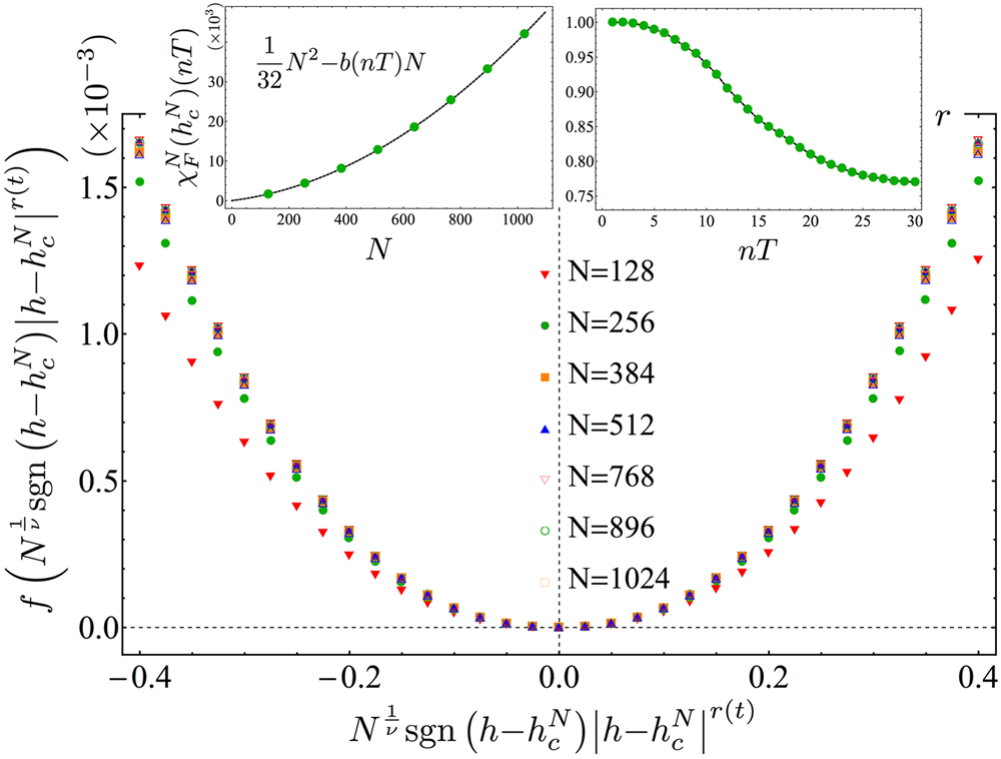
Finite-size scaling of the Fidelity susceptibility. Main plot: Data collapse of the Fidelity susceptibility according to the Ansatz in Eq. 4 after *n* = 15 periods, with exponents *r*(15*T*) = 0.86 and $\nu =1$. Left inset: The maximum of the Fidelity susceptibility attained at the pseudocritical point ${\chi }_{F}^{N}({h}_{c}^{N})$ after *n* = 15 driving periods. The plot shows an algebraic divergence with the system size. Right inset: Values of the time-dependent exponent *r*(*nT*) that allows for the FSS data collapse.

Let us now turn our attention to the Fidelity susceptibility (FS). The ground state FS is defined by $|\langle GS(h)|GS(h+\delta h)\rangle |$ and it depends on three length scales; namely, the system size *N*, the correlation length $\xi \sim {|h-{h}_{c}|}^{-\nu }$ and the length scale associated to the parameter *δh*, ${\xi }_{\delta h}\sim {|\delta h|}^{-\nu }$. If ${\xi }_{\delta h}$ is the largest length scale, it is meaningful to consider the FS^56, 57^, defined by ${\chi }_{F}^{N}(h)=-\frac{{\partial }^{2}F}{\partial {(\delta h)}^{2}}$, whose scaling behaviour will be dictated by the other two length scales (for a detailed analysis of the use FS in transverse field spin models see the book by Dutta *et al*. in ref. 50). For the Ising model at equilibrium, it has been shown that, at criticality, where $\xi \gg N$, the FS exhibits a maximum whose height scales algebraically with the system size as ${\chi }_{F}^{N}({h}_{c}^{N})=\frac{1}{32}({N}^{2}-N)$. Far from criticality, on the other hand, where $\xi \ll N$, the scaling is extensive^58, 59^. In the following we will investigate the former limit as the driving is around criticality. For the analysis of the stroboscopic Fidelity susceptibility in the limit where $\xi \ll N$, see the Section Methods. Notice that, contrary to the quantities considered before, ${\chi }_{F}^{N}$ does not scale logarithmically with *N*. Notwithstanding, FSS is still attainable, provided a new, time-dependent exponent is introduced, which takes into account the fact that the algebraic scaling gets modified. At equilibrium, one considers the susceptibility of the ground state fidelity, ${F}^{N}(h)=|\langle GS(h)|GS(h+\delta h)\rangle |$ for *δh* → 0, which, by definition, is time-independent and can be related also to the irreversible work in an infinitesimal quench protocol^60^. On the other hand, in the presence of the driving, we will consider the susceptibility of the following expression for the Fidelity: ${F}^{N}(h)(nT)=|\langle GS(h)|{\hat{U}}^{\dagger }(nT)\hat{U}^{\prime} (nT)|GS(h+\delta h)\rangle |$, where $\hat{U}$ and $\hat{U}^{\prime}$ differ as they correspond to driving around *h* and *h* + *δh*, respectively. ${F}^{N}(h)(t=nT)$ reduces to the ground state Fidelity at *t* = 0. In Fig. 2, we report our results for the fidelity susceptibility, ${\chi }_{F}^{N}$, obtained by taking *δh* = 10^−5^ and the same driving parameters as in Fig. 1. Interestingly enough, we find that the algebraic divergence is preserved, according to the law ${\chi }_{F}^{N}({h}_{c}^{N})(nT)=\frac{1}{32}{N}^{2}-b(nT)N$, where *b*(*nT*) ($b\mathrm{(0)}=\tfrac{1}{32}$) is a monotonically increasing function (see left inset in Fig. 2). This behaviour can be qualitatively explained by noticing that the driving ultimately will invalidate the FSS behaviour (hence a linear scaling with *N* is retrieved) at later times (see following subsection). As a consequence, we modify the FSS Ansatz^61^, by introducing a time-dependent exponent *r*(*nT*) (*r*(0) = 1),

4 $$\begin{array}{c}\frac{{\chi }_{F}^{N}({h}_{c}^{N})(nT)-{\chi }_{F}^{N}(h)(nT)}{{\chi }_{F}^{N}(h)(nT)}=f\,({N}^{\frac{1}{\nu }}\,{\rm{sgn}}\,(h-{h}_{c}^{N}){|h-{h}_{c}^{N}|}^{r(nT)}),\end{array}$$

where sgn(·) is the sign function. In the main plot of Fig. 2 we show an instance of the data collapse obtained by means of Eq. 4 after *n* = 15 periods, where *r*(*nT*) = 0.86. By choosing a different number of periods, the exponent *r*(*nT*) decreases following the curve reported in the right inset of Fig. 2. It is worth mentioning that our *r*(0) = 1 coincides with the equilibrium value, which is given by $r\mathrm{(0)}=\frac{{d}_{a}^{c}-{d}_{a}}{\nu }$, where ${d}_{a}^{c}=2$ and *d*_*a*_ = 1 are the critical adiabatic dimension and the adiabatic dimension, respectively^61^. The *d*’s cannot be easily defined out-of-equilibrium, and, therefore, we relied on numerical tools to extract the best collapse exponent *r*(*nT*). Nevertheless, notice that the critical exponent $\nu$ equals 1 at all times where the FSS holds.

Notably, all the quantities whose FSS we have reported so far for the Ising model (*γ* = 1 in Eq. 1), maintain this scaling behaviour, with the same critical exponents, in the whole XY universality class 0 < *γ* ≤ 1.

### Breakdown time of finite-size scaling

The FSS under periodic driving holds up to a characteristic time *τ*_*bd*_(*N*), which we dub *breakdown time*, and which depends on the driving parameters. Remarkably, we obtain for $\omega  > 4$ and ${\rm{\Delta }}h\ll {h}_{c}$, a breakdown time comparable to the recurrence time ${\tau }_{bd}(N)\simeq {t}_{rec}$ (where ${t}_{rec}=N/\mathrm{(2}{v}_{max})$), while for smaller frequencies, the breakdown time occurs before the onset of recurrences (*τ*_*bd*_ < *t*_*rec*_). Hence, for a large frequency and small amplitude driving, the breakdown of critical FSS could be arbitrarily delayed by taking systems of a large-enough-size. For the high-frequency limit, see also a recent paper by Gritsev and Polkovnikov^62^, where it has been shown that the Floquet Hamiltonian of a step-like periodically driven Ising model shares the same critical properties of the time-independent Ising model based on the structure of the Onsager algebra and the self-duality (in the limit of $\omega \to \infty$, the system freezes in its initial state and FSS is attainable for every *n* and *δh*, but this would fall us back into the equilibrium scenario).

**Figure 3:**
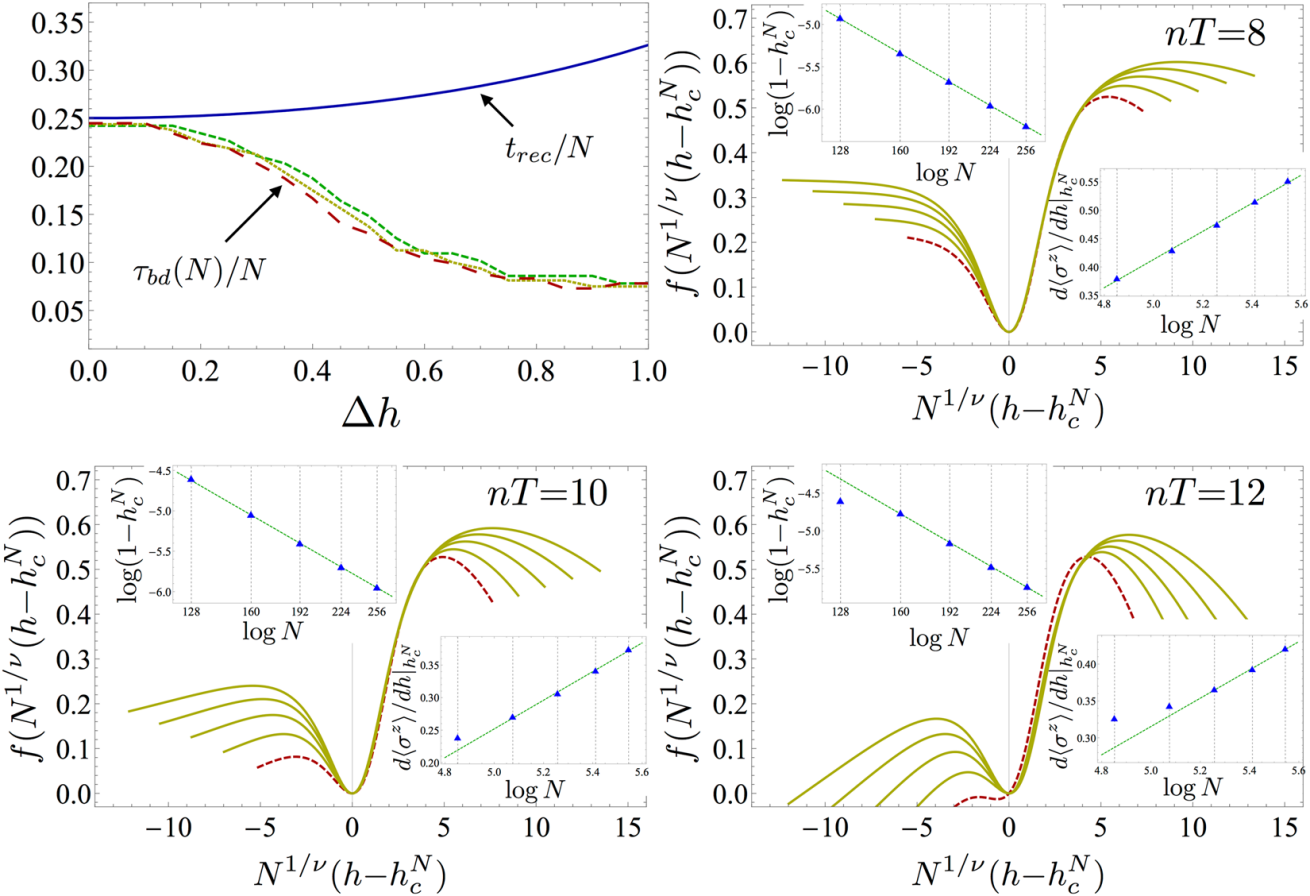
Breakdown of finite-size scaling. (Upper left) Maximum number of cycles for which FSS holds for the *z*-susceptibility, with different system sizes, *N* = {128, 160, 192}, as a function of the applied amplitude for a driving frequency $\omega =4$ in the Ising model (*γ* = 1 in Eq. 1). Notice the relation ${N}_{2}{\tau }_{bd}({N}_{1})\simeq {N}_{1}{\tau }_{bd}({N}_{2})$, where *N*_1_ and *N*_2_ are different system sizes, indicating that the breakdown time scales linearly with *N*. The continuous blue line shows the recurrence time *t*_*rec*_ and one sees that, for driving amplitudes Δ*h* < 0.1, ${\tau }_{bd}\simeq {t}_{rec}$. In the other three panels, we report an instance of the breakdown of FSS for driving parameters $\omega =4$ and Δ*h* = 0.75 in the Ising model, taken after *n* = 8, 10, and 12 driving periods, respectively, with *N* = {128, 160, 192, 224, 256}. We see that the curves corresponding to lower *N* depart from the FSS Ansatz more and more with increasing *n*, while data collapse is still achieved for the other sizes. The insets in each figure report the algebraic fit of the pseudocritical point ${h}_{c}^{N}$ (upper left inset) and the logarithmic divergence of the maximum of the magnetic susceptibility ${\chi }_{z}^{(N)}({h}_{c}^{N})$, showing that, at the breakdown time *τ*_*bd*_(*N*), only the points corresponding to the lower *N*’s do not satisfy the respective scaling relation.

On the other hand, the breakdown of FSS at times smaller than *t*_*rec*_ that occurs for small frequencies ($\omega  < 4$) is mostly due to the fact that systems with small sizes loose their scaling properties. As an instance of such a behaviour, in Fig. 3 we display *τ*_*bd*_(*N*), as extracted from the transverse magnetisation susceptibility. In the upper left panel, we show that the breakdown time is comparable to the recurrence time for small driving amplitudes; conversely, by increasing Δ*h*, the number of cycles for which FSS holds reduces significantly, as can be expected by observing that already in the first few cycles a considerable amount of energy is absorbed by the system. In the same figure, we report three instances of scaling analysis performed at increasing number of cycles. FSS holds for all cases but the one corresponding to the system of smaller size, so that all of the curves collapse near *h*_*c*_, but one.

**Figure 4:**
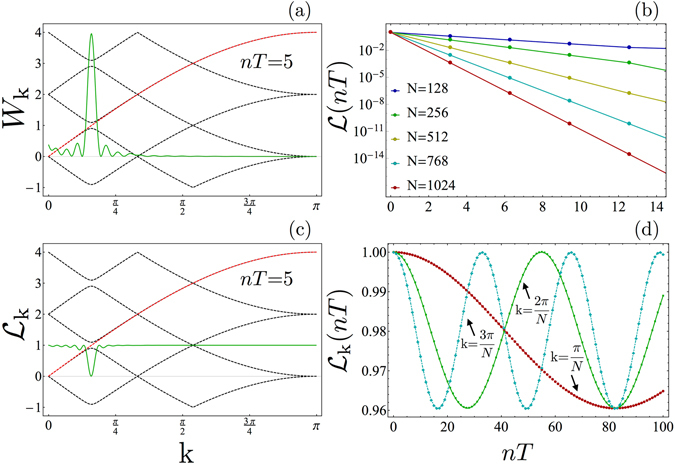
Loschmidt echo. (**a**) Work performed by the driving agent after *t* = 5*T*, resolved in *k*: modes near the quasi degeneracy in the Floquet spectrum absorb much more than the others. (**b**) Loschmidt echo evaluated at the stroboscopic times, showing an exponential decay. (**c**) Momentum resolved Loschmidt echo evaluated at the fixed time *t* = 5*T*, showing that modes near the quasi-degeneracy give the dominant contribution to the decay of ${\mathcal L}$. (**d**) Stroboscopic time evolution of the *k*-resolved Loschmidt echo, for the longer wavelength modes, showing an oscillatory behavior. All the plot are drawn for a driving with $\omega =2$ and Δ*h* = 0.1. The two left panels (a,c) also report the Floquet spectrum (dashed black lines) and the single-particle energies of the unperturbed Hamiltonian Eq. 2 (red line) on the background.

We stress that the time scales for which FSS persists are well beyond the very short transient where the system remains in the close neighborhood of its initial critical equilibrium state. Indeed, by evaluating the Loschmidt Echo (see Methods), ${\mathcal L} (nT)=|\langle {\rm{\Psi }}(nT)|GS\rangle |$, which gives the probability amplitude to find the system in a state close to the initial critical ground state^63^, we find–already after a few cycles–that ${\mathcal L} (nT)$ has become negligibly small. This is explicitly shown in Fig. 4(b), where an exponential decay of ${\mathcal L}$ is reported. A closer look, however, shows that the decay of the Loschmidt echo is essentially due to those mode *k* for which a quasi-degeneracy occurs in the Floquet energies^28^. These almost degenerate modes are also those responsible for energy absorption from the driving, see Fig. 4.

Actually, the Loschmidt echo can be decomposed as a product of contributions from the different modes, ${\mathcal L} ={\prod }_{k}\,{ {\mathcal L} }_{k}$ (see the methods section). Such a momentum resolved Loschmidt echo is shown in Fig. 4(c), where it is superimposed to the dispersion relation of the Floquet quasi energies (given by the black dashed lines on the background). One can see that the minimum of ${ {\mathcal L} }_{k}$ is found where an ‘inter-band quasi-degeneracy’ occurs. Moreover, this ‘quasi-resonance’ precisely corresponds to the peak of the energy absorbed from the driving, as shown in Fig. 4(a). There, the amount of work performed by the driving agent is reported for each mode *k* (see Methods). Therefore, we can conclude that the decay of the Loschmidt echo is due to these specific modes, where a Floquet resonance occurs, and which are brought significantly out of equilibrium by the absorption of energy from the driving.

On the other hand, one expects FSS to be essentially a feature of the long wavelength (and low energy) modes. These modes are much less affected by the driving as they absorb much less energy than those close to the quasi degeneracy. Correspondingly, their contribution to the overall decay of the Loschmidt echo is very small. The time dependence of the *k*-resolved Loschmidt echo for such small-*k*-modes is reported in Fig. 4(d), where we see that ${ {\mathcal L} }_{k}(nT)$ periodically oscillates in time. For each k, the period of such oscillations is determined by the k-eigenvalue of ${\hat{H}}_{F}$ (i.e. $\hslash /{\mu }_{k}$ see Methods), which becomes larger and larger with increasing *N*.

It turns out that the breakdown of FSS occurs close to the time where a minimum is found for the ${ {\mathcal L} }_{k}$ corresponding to the smallest *k*. Therefore, ${\tau }_{bd}(N)\approx \hslash /\mathrm{(2}{\mu }_{k})$. After this time, in fact, it not possible anymore to obtain the collapse of curves corresponding to physical observables evaluated for different *N*’s. This is due to the fact that systems with different sizes behave asynchronously. This means that, although the ${ {\mathcal L} }_{k}$ corresponding to the smallest *k* goes back to unity after a period, this revival occurs at a time that explicitly depends on the system size. As a result, since the scaling is a comparison of physical quantities for different *N* at the same time, it does not hold anymore because systems with different sizes have *k*-resolved Loschmidt echoes that are ‘out of phase’ from each other.

Finally, let us comment on the fact that for low-$\omega$ drivings, the FSS behaviour is lost already after a single cycle because the low-*k* modes absorb more energy from the drive as the Floquet resonances move towards them, (see Methods).

## Discussion

In summary, we have shown that the scaling behaviour proper of an equilibrium quantum critical point retains its validity also when the system is brought out-of-equilibrium via a periodic drive. Finite-size scaling of both local and global quantities, exhibiting logarithmic as well as algebraic scaling with the system size, has been performed. We have shown that the equilibrium critical exponents are robust against the periodic perturbation up to times when the stroboscopic state is far from the quantum critical state, suggesting that the features of universality manifest themselves also under strong periodically driven settings. In addition, our claims are within reach of experimental verification, as out-of-equilibrium quantum Ising dynamics is currently under active investigation via a great variety of different physical systems, ranging from degenerate Bose gases in a optical lattice^64, 65^ to Rydberg atoms^66^. Our results may find applications also in the emerging field of out-of-equilibrium quantum thermodynamics, where, recently^67^, quantum Otto engines, having as working substance a many-body system at the verge of criticality, have been suggested to be able to attain the Carnot efficiency at finite power because of the validity of the FSS relations.

It would be interesting to study in the future whether the same scenario holds for non-integrable systems hosting a quantum phase transition, as, for instance, the one dimensional Bose-Hubbard model, where, however, the system is eventually driven into an infinite temperature state, and therefore the persistence of critical scaling is expected only in a temporal window delimited by the thermalization time of the system. A different scenario, on the other hand, could emerge for interacting integrable models, such as the antiferromagnetic XXZ Hamiltonian, where the Néel and the XY phase are separated by a second-order QPT and thermalisation is prevented by the integrability of the model.

## Methods

### Concurrence

The concurrence, *C*(*ρ*), is a measure a bipartite qubit entanglement, which can be straightforwardly computed for any two-spin−$\tfrac{1}{2}$ density matrix *ρ* ^37^. In particular, we apply it to evaluate the entanglement between two spins of the chain, residing at sites *i* and *j*. In this case, the two spin reduced density matrix, ${\hat{\rho }}_{i,j}$, is obtained by computing the partial trace over all but the *i*-th and *j*-th spin degrees of freedom of either (i) the ground state |*GS*〉, or (ii) the stroboscopical state $|{\rm{\Psi }}(nT)\rangle$. Given the state, *C*_*i*,*j*_ can be evaluated, once the state ${\hat{\rho }}_{i,j}$ is expressed in the logical basis of the eigenstates of the ${\hat{\sigma }}^{z}$ operator, via the relation $C=\,{\rm{\max }}\,[0,\sqrt{{\lambda }_{1}}-{\sum }_{n=2}^{4}\,\sqrt{{\lambda }_{n}}]$, where the *λ*_*n*_ are the eigenvalues, in decreasing order, of $\hat{\tilde{\rho }}=({\hat{\sigma }}^{y}\otimes {\hat{\sigma }}^{y})\,{\hat{\rho }}^{\ast }\,({\hat{\sigma }}^{y}\otimes {\hat{\sigma }}^{y})$.

### Loschmidt echo

The Loschmidt echo is defined as

$$ {\mathcal L} (nT)=|\langle {\rm{\Psi }}(t=0)|{\rm{\Psi }}(nT)\rangle |.$$

Using the explicit form of the initial and stroboscopic state, namely

$$|{\rm{\Psi }}\mathrm{(0)}\rangle =\prod _{k > 0}\,({u}_{k}\mathrm{(0)}+{v}_{k}\mathrm{(0)}{\hat{c}}_{k}^{\dagger }{\hat{c}}_{-k}^{\dagger })\,|0\rangle ,$$

$$|{\rm{\Psi }}(nT)\rangle =\prod _{k > 0}\,({u}_{k}(nT)+{v}_{k}(nT){\hat{c}}_{k}^{\dagger }{\hat{c}}_{-k}^{\dagger })\,|0\rangle ,$$

we obtain ${\mathcal L} (nT)={\prod }_{k}\,{ {\mathcal L} }_{k}(nT)$, where the *k*-resolved Loschmidt echo is given by

$${ {\mathcal L} }_{k}(nT)=|{u}_{k}\mathrm{(0)}{u}_{k}(nT)+{v}_{k}\mathrm{(0)}{v}_{k}(nT)|.$$

For an extensive analysis of the Loschmidt echo in periodically driven systems see ref. 63.

### Work

The (average) work performed up to time *t* by driving the system is given by the difference between the average instantaneous energy of the system and its initial value given by the ground state energy. At the discrete time instants *t* = *nT*, we have

$$W(nT)=\langle {\rm{\Psi }}(nT)|\hat{H}\mathrm{(0)}|{\rm{\Psi }}(nT)\rangle -{E}_{GS},$$

where we used the fact that $\hat{H}(nT)=\hat{H}\mathrm{(0)}$.

Using the explicit expression of $\hat{H}$ in terms of the Bogoliubov fermions, Eq. 2, we have that the constant terms cancel out and that the work naturally decomposes into the sum of contributions arising from each mode *k*,

$$W(nT)=\sum _{k}\,{W}_{k}(nT),$$

where

$${W}_{k}(nT)=2{\varepsilon }_{k}\,\langle {\rm{\Psi }}(nT)|{\hat{\gamma }}_{k}^{\dagger }{\hat{\gamma }}_{k}|{\rm{\Psi }}(nT)\rangle .$$

Notice that, since the Hamiltonian undergoes a periodic driving, and we are evaluating the work at an integer number of periods, the average work coincides in our case with both the so called irreversible work and the inner friction^25^, so that it can be used to describe also the amount of irreversibility brought into the system.

### Floquet spectrum

The time-independent effective Hamiltonian, dubbed *Floquet* Hamiltonian ${\hat{H}}_{F}$ and corresponding to the time-dependent Ising Hamiltonian in Eq. 1 of the main text, can be expressed in quadratic form ref. 27 as,

5 $${\hat{H}}_{F}=\sum _{k > 0}\,{\hat{h}}_{kF}=\sum _{k > 0}\,{\mu }_{k}^{+}{({\hat{\mu }}_{k}^{+})}^{\dagger }{\hat{\mu }}_{k}^{+}+{\mu }_{k}^{-}{({\hat{\mu }}_{k}^{-})}^{\dagger }{\hat{\mu }}_{k}^{-},$$

where $\{{\mu }_{k}^{\pm },|{\mu }_{k}^{\pm }\rangle \equiv {({\hat{\mu }}_{k}^{\pm })}^{\dagger }|0\rangle \}$ are, respectively, the positive and negative Floquet eigenvalues and eigenvectors of the Floquet Hamiltonian ${\hat{h}}_{kF}$ for the mode *k*. The evolution operator ${\hat{U}}_{k}(T)={e}^{-iT{\hat{h}}_{kF}}$ is determined by the solution of the Bogoliubov-de Gennes equations

6 $$i(\begin{array}{c}{\dot{u}}_{k}(t)\\ {\dot{v}}_{k}(t)\end{array})=(\begin{array}{cc}\cos \,k-h(t) & \sin \,k\\ \sin \,k & -\cos \,k+h(t)\end{array})\,(\begin{array}{c}{u}_{k}(t)\\ {v}_{k}(t)\end{array}),$$

for each mode *k* with the initial condition $\{{v}_{k}\mathrm{(0)},{u}_{k}\mathrm{(0)}\}=\{0,1\}$ after one period *t* = *T*. Because of the periodicity of the Hamiltonian in Eq. 1 of the main text, the Floquet eigenvectors are defined up to a periodic phase, corresponding to a shift of the eigenenergies of an integer multiple of the driving frequency $\omega$, ${\mu }_{k}^{\pm }\to {\mu }_{k}^{{\pm }^{(l)}}={\mu }_{k}^{\pm }+l\omega$. The latter symmetry brings to the definition of the Brillouin zones $BZ(l)=-[(l-\mathrm{1)}\frac{\omega }{2},l\frac{\omega }{2})\cup [(l-\mathrm{1)}\frac{\omega }{2},l\frac{\omega }{2})$ as those reported in Fig. 4 in the main text. As a consequence, resonances can occur both within the same band and between different bands, dubbed intra-band and inter-band resonances, respectively, in the main text.

Finally, as the stroboscopic evolution of the initial state is given by $|{\rm{\Psi }}(nT)\rangle ={e}^{-iT{\hat{H}}_{F}}|{\rm{\Psi }}\mathrm{(0}\rangle$, the dynamics is governed by the Floquet quasiparticles energies *μ*_*k*_ and hence the maximum velocity quasiparticles can spread out is given by ${v}_{max}=\mathop{{\rm{\max }}}\limits_{k}\frac{d{\mu }_{k}}{dk}$, that is the maximum of the “group velocity” as given by the “dispersion relation” of ${\hat{H}}_{F}$.

### Extensive scaling of the Fidelity susceptibility far from criticality

**Figure 5:**
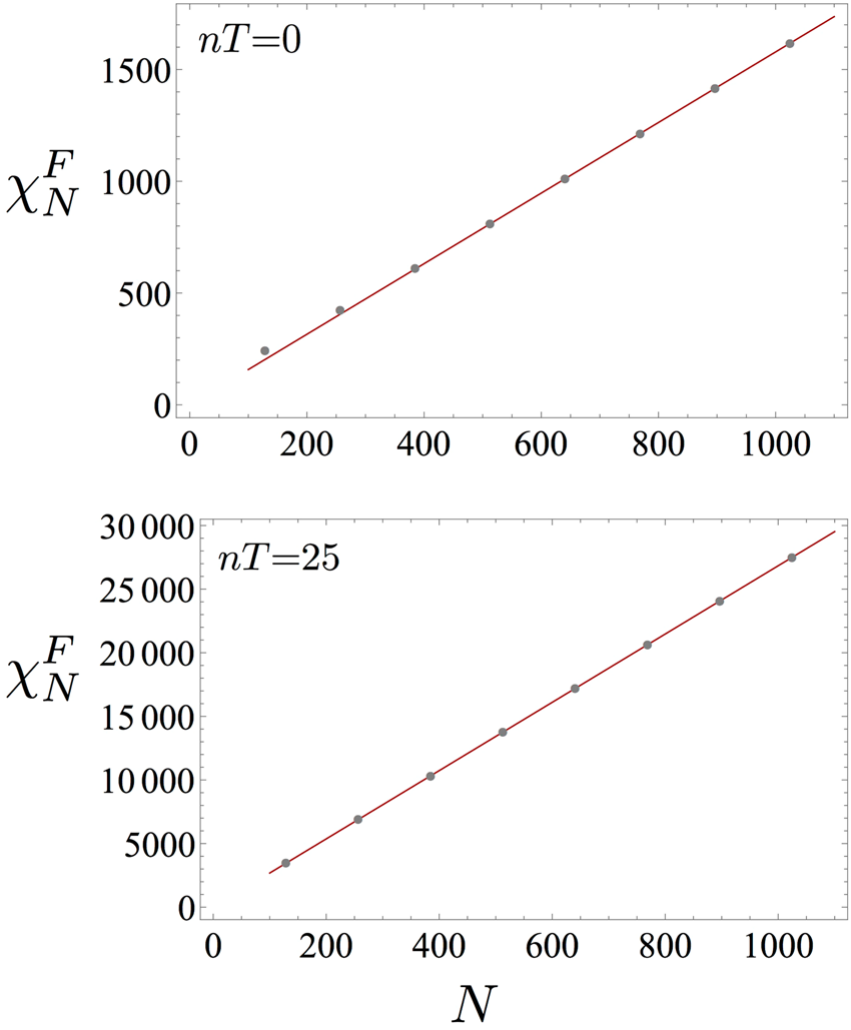
Non-critical linear scaling of the fidelity susceptibility. (upper plot) Linear scaling of the fidelity susceptibility at equilibrium in the region where $N\gg \xi$. (lower plot) The linear scaling is preserved also after *n* = 25 cycles of the periodic drive. The dynamical parameters in both plots are: *h*(0) = 0.95 and *δh* = 10^−5^ in the fidelity *F* ^*N*^(*h*) of the main text, and, in addition, $\omega =2\pi$, and Δ*h* = 0.1 for the stroboscopic fidelity ${F}^{N}(h)(nT)$.

In the main text we have investigated the fidelity susceptibility (FS) in the limit where the order parameter correlation length $\xi \sim {|h-{h}_{c}|}^{-\nu }$ dominates over the system size *N*, i.e., $\xi \gg N$. In such a regime, the FS scales as *N* ^2^ at equilibrium and FS finite-size scaling has been reported in Fig. 2 of the main text. On the other hand, if $N\gg \xi$, the FS scales at equilibrium linearly with *N*. Here we will show that such a linear scaling is preserved also under periodic drive. In Fig. 5 we report the fidelity susceptibility both at equilibrium and after *n* = 25 cycles of the driving.

**Figure 6:**
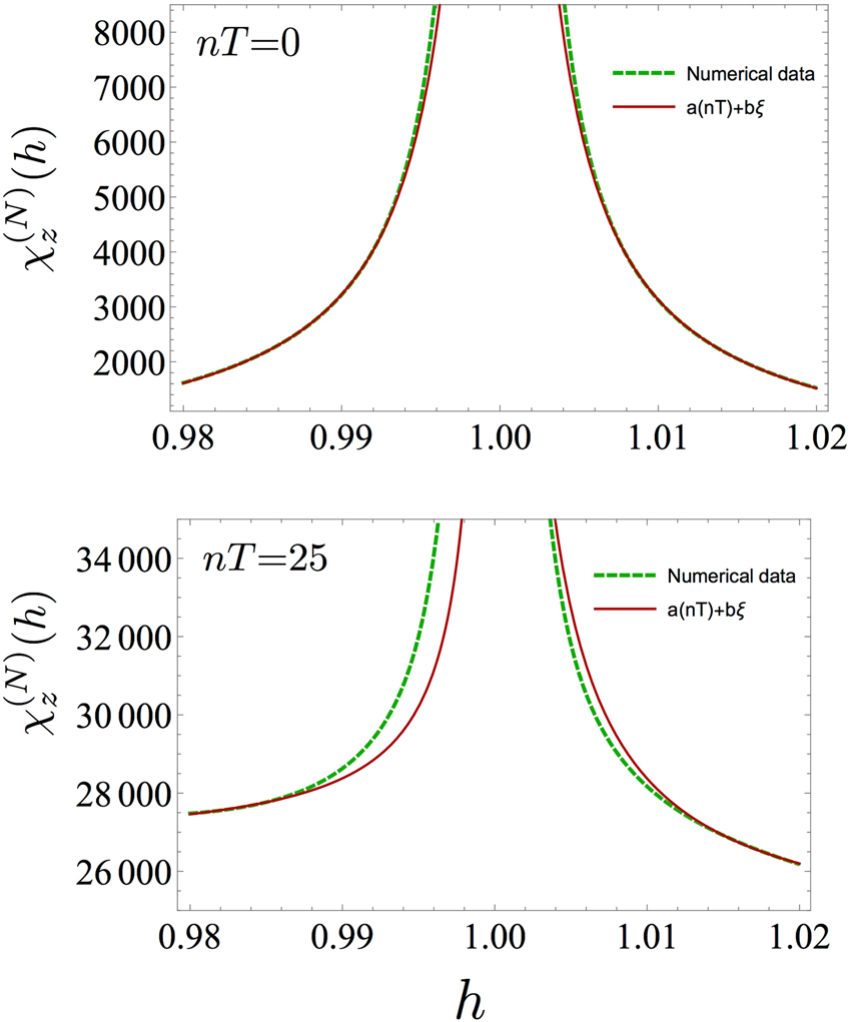
Scaling of the fidelity susceptibility with the magnetic field. Fidelity susceptibility ${\chi }_{z}^{(N)}(h)$ in the limit $N\gg \xi$ in the paramagnetic (*h* > 1) and ferromagnetic phase (*h* < 1) at equilibrium (upper plot) and after *n* = 25 cycles (lower plot). The fitting curve ${\chi }_{z}^{(N)}(h)=a(nT)+b\,\xi$, where $\xi ={(\mathrm{ln}h)}^{-1}$ accurately overlaps with the numerical results at *nT* = 0, as expected away from criticality. At *n* = 25, the scaling of ${\chi }_{z}^{(N)}(h)$ as ${(\mathrm{ln}h)}^{-1}$ is still visible, although the range of validity has decreased to points further away from criticality than in the equilibrium case. Both plots are reported for *N* = 1024, *δh* = 10^−5^, $\omega =2\pi$, and Δ*h* = 0.1 in the driving protocol.

Furthermore, by fixing *N*, in the limit $N\gg \xi \sim {(\mathrm{ln}h)}^{-1}$, i.e., away from criticality, it is known that at equilibrium the fidelity susceptibility ${\chi }_{z}^{(N)}(h)$ scales as $\xi$ ^50^. In Fig. 6 we report, for *N* = 1024, ${\chi }_{z}^{(N)}(h)$ as a function of *h* for drivings both in the ferromagnetic and the paramagnetic phase. By considering values of *h* such that the correlation length fulfills $\xi \ll N$, we notice that the scaling of ${\chi }_{z}^{(N)}(h)$ as $\xi$ is preserved also under periodic drive, although the range of validity of such a scaling shrinks by increasing the number of periods *n*. Nevertheless, a fitting curve of the type ${\chi }_{z}^{(N)}(h)=a(nT)+b\,\xi$ overlaps with our numerical results still after *n* = 25 periods of the driving for values of *h* further away from criticality. Notice also that the fitting curve has a time-dependent coefficient, *a*(*nT*), that plays the same rôle of *b*(*nT*) for the FSS behaviour of ${\chi }_{z}^{(N)}(h)$ at criticality, see Fig. 2.

### Low-*ω* drivings

**Figure 7:**
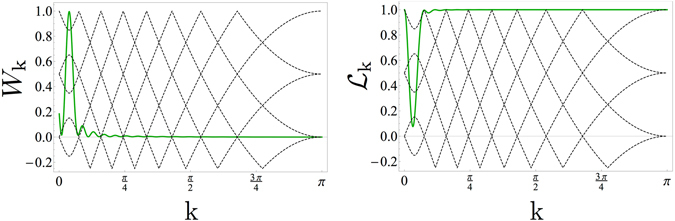
Low-frequency drive. (upper panel) Work performed by the driving agent after *t* = *T*, resolved in *k*: the resonances in the Floquet spectrum move towards the low-*k* region of the spectrum of the unperturbed Hamiltonian $\hat{H}\mathrm{(0)}$. (lower panel) Momentum resolved Loschmidt echo evaluated at the fixed time *t* = *T*, showing that, for the modes near the quasi-degeneracy, now in the FSS-relevant region, the decay of the Loschmidt echo is both faster and more significant than for higher $\omega$ (see Fig. 4 in the main text). In both plots we considered a driving frequency $\omega =0.5$.

In the main text of the manuscript we have investigated mainly drivings at frequencies $\omega  > 4$, where the FSS behaviour is resilient under the periodic modulation of the magnetic field *h*(*t*). In this subsection we show that, in the low-frequency limit, the FSS is lost already after the first cycle. To support this claim, we report in Fig. 7 the *k*-resolved work and Loschmidt echo for $\omega =0.5$. As already stated in ref. 28, by decreasing the frequency of the drive, the number of resonances in the Floquet spectrum increases and, more importantly, they also move towards the low *k*-modes. As a consequence, the energy injected into the system by the low-frequency driving is absorbed mainly by the latter and also the Loschmidt echo of the low-*k* modes decreases to values significantly lower than in the $\omega  > 4$ case. As a result, the FSS behaviour is not resilient to such low-$\omega$ drives.
